# Bromide of Potassium and the Diseases of Dentition

**Published:** 1873-09

**Authors:** C. G. Polk


### ARTICLE V.

### *Bromide of Potassium and the Diseases of Dentition.*

BY C. G. POLK, M. D.

As Bromide of Potassium has been applied to nearly every disease to which flesh is heir, anæmia and gastritis perhaps not excepted, I supposed, until recently, that the value of this agent in the disorders of dentition was well understood, and that it was frequently used. Finding the application of it to that class of disorders quite a novelty to many, I will give my experience, hoping to awaken attention to it from those possessing larger fields of observation, to verify or disprove my conclusions.

I have used Bromide of Potassium in about one hundred cases of infantile diseases, embracing those of diarrhœa, pulmonary congestion, and cerebral congestion, arising from dental irritation. I have seen the diarrhœa which had defied chalk, acetate of lead, calomel, catechu, opium, the sulphites, and pepsin, yield in forty-eight hours to this Bromide. I have seen persistent vomiting, which is often in such cases, nought else but reflex action from the brain, cease after a full dose of this agent. I have seen the hot head, with gritting of the teeth, presages of convulsions, yield to a few doses. I have seen pulmonary congestion, dependent on the same cause, yield in a few days.

The most marked case I recollect was a daughter of Mr.

Pickett, of Frankford Arsenal. I had diarrhœa and both cerebral and pulmonary congestion to contend with, and at the time I resorted to the Bromide of Potassium, the case seemed hopeless; under its influence she made a rapid recovery.

The dose must be adapted to each case.—*Med. and Surg. Reporter.*

---

